# Getting Back on Track: Meanings of Recovery After Critical Illness Caused by COVID-19

**DOI:** 10.1177/23779608241282922

**Published:** 2024-10-14

**Authors:** Päivi Juuso, Åsa Engström, Ulrica Strömbäck, Maria Andersson, Anna Nordin

**Affiliations:** 1 Division of Nursing and Medical Technology, Department of Health, Education and Technology, 91874 Lulea University of Technology, Luleå, Sweden; 2 Faculty of Health, Science, and Technology, Department of Health Science, 4209 Karlstad University, Karlstad, Sweden

**Keywords:** COVID-19, critical illness, nursing, phenomenological hermeneutic interpretation, qualitative design, recovery

## Abstract

**Introduction:**

Being critically ill in need of intensive care, lead to a challenging way back after survival, so also for survivors of COVID-19. The process to recovery can be long.

**Objectives:**

The aim of our qualitative study was to elucidate meanings of recovery for people who were once critically ill with COVID-19.

**Method:**

We conducted qualitative individual interviews with 13 individuals who had been critically ill with COVID-19, following a narrative approach. The data collected from the interviews, were analyzed according to phenomenological hermeneutic interpretation.

**Results:**

The participants, although feeling alone in the process of recovery, had willpower to return to normal life but struggled to keep pace with others. They strived for balance in everyday life and to regain strength despite being exhausted after having COVID-19. The participants were grateful for their survival but displayed a need to understand what had happened. They longed for social contact, expressed feelings of abandonment, and wished for follow-up dialogues with healthcare professionals to better understand their situation. However, because support from healthcare was insufficient, the participants ultimately needed to develop their own strategies to cope with their questions, fears, and weakness.

**Conclusion:**

Meanings of recovery for people once critically ill with COVID-19, is to strive for balance in everyday life. In their recovery process, healthcare professionals should seek to understand what the illness means for the ill person, and in mutual understanding support them based on their needs.

## Introduction

On March 11, 2020, the World Health Organization (2020a, 2020b) declared the outbreak of the coronavirus disease 2019 (COVID-19) to be a pandemic and a public health emergency.

During the phases of the pandemic, the authorities in Sweden took several measures to mitigate the effects on the population. Instead of strict lockdowns, the Swedish approach to the COVID-19 pandemic relied on voluntary measures, protection of high-risk groups, and keeping primary schools open. At the time of the interviews of this study (July 2020–March 2021), temporary laws and restrictions were passed, and Swedish authorities encouraged people to work from home where possible and to avoid public transportation. Social gatherings were limited and to protect vulnerable populations, especially people aged 70 or older were recommended to limit social contact, and those with COVID-19 symptoms were directed to self-isolate (HSLF-FS 2021:104). Later on, strict laws and restrictions were passed to prevent spread of the disease (SFS 2021:4). In this study, the focus is on meanings of recovery for people once critically ill in COVID-19.

## Review of Literature

Millions of people became critically ill during the COVID-19 pandemic and required hospitalization and intensive care (CDC COVID-19 Response Team, 2020). Of these, a considerable proportion developed acute respiratory distress syndrome (Grasseli, Greco, et al., 2020; Grasseli, Zangrillo, et al., 2020) and required extended periods of invasive mechanical ventilation (Richardson et al., 2020), shown to be correlated with a higher risk of muscle weakness (Medrinal et al., 2021). Many patients with COVID-19 also required increased oxygenation in prone positioning (Langer et al., 2021) which can lead to complications such as musculoskeletal injuries (Binda et al., 2022; Goettler et al., 2002; Malik et al., 2020).

Although the number of survivors of COVID-19 admitted to intensive care units (ICUs) continued to increase, studies showed that their health status was worse than others without the infection one year after discharge from hospital (Huang et al., 2021) and their experiences with the disease had significantly impacted their quality of life (Gamberini et al., 2021; Nandasena et al., 2022). Shang et al. (2021) highlighted that sequela such as fatigue, sleep disorder, and shortness of breath were common up to six months after recovery.

COVID-19 symptoms affecting the respiratory, musculoskeletal, psychological, neurocognitive, and gastrointestinal systems, among others, have led to long-term consequences for patients once critically ill (Pierce et al., 2022; Swedish Agency for Health Technology Assessment and Assessment of Social Services [SBU], 2022). Patients once critically ill in COVID-19 have shown impaired lung function and lung damage, cardiovascular effects, cerebral changes, and an impaired sense of smell and taste (SBU, 2022). COVID-19 had also adverse effects on patients with extensive medical histories for example oncology patients. The social, economic, and mental health effects of the pandemic affected the patient's quality of life, survival, and their healing process (Fadavi et al., 2021).

Being critically ill with COVID-19 has been described as a fight against an invisible enemy and as living in a nightmare (Engström et al., 2022). For patients, recovering from COVID-19 involves feelings of alienation and the need to feel safe, experience a sense of familiarity, and recognize oneself. A recent study shows that patients recovering from COVID-19 perceive elevated levels of illness-related uncertainty and stress (Nabi Foodani et al., 2024). Physical weakness and a lack of bodily control have also been described by patients recovering from COVID-19, some of whom found the experience to be terrifying (van Oorsouw et al., 2022). Simultaneously, patients have also reported experiencing fear from memories or symptoms, like those before falling ill, as well as fighting to achieve control and to stabilize their lives (Vogel et al., 2021). Diaries written by the healthcare professionals during the patient's admission at ICU has been described as helping patients to connect to their memories about what happened during their time at ICU (Barreto et al., 2019).

Goodwin et al. (2021) found that early mobilization and post-ICU rehabilitation enhanced recovery among people with severe respiratory illnesses, including COVID-19, which agrees with Parker et al.'s (2021) findings on post-COVID-19 experiences. Here, the majority of patients (83%) had complete resolution of radiographic findings and normal oxygen saturations both at rest and after exertion. However, a reduction in grip strength was identified as well as clinically significant post-traumatic stress symptoms in a majority (57%) of the patients.

According to Kean et al. (2016), the process of recovering from critical illness is mostly about developing a sense of survivorship in a series of physical, psychological, and social transitions. It is an active process in which survivors engage with their postillness selves which demands ICU survivors to actively engage and contribute their experiences, knowledge, and aspirations. Friedman (2021) too advocates for recovery as a process in which the patient, instead of coming back to the same state of soundness as before illness or malady, moves forward a new state of soundness. The many ways in which the patients can recover is also reflected in the use of the term recovery processes which is suggested. Although they still possess issues from either the condition itself or the treatment, survivors after the COVID-19 are considered cured, displaying the complexity in recovery processes. Because studies on the recovery experiences of people once being critically ill with COVID-19 remain scarce, the aim of our study was to elucidate meanings of recovery for those patients.

## Method

### Design

In our study, we adopted a qualitative design and, to collect data, we conducted individual interviews following a narrative approach, as the aim was to understand the meanings emerging during the interviews (Mishler, 1986). To reach a deeper understanding of the phenomena, that is, meanings of recovery, we analyzed the data according to phenomenological hermeneutical interpretation (Lindseth & Norberg, 2004).

### Participants and Inclusion Criteria

All prospective participants (*n *= 17), recruited via purposive sampling, based on the inclusion criteria that they had been critically ill with COVID-19 during the first surge of the pandemic, and had been admitted to a COVID-19-specific ICU in the northern-most part of Sweden. Once contacted by the head of the ICU and informed about the study and its aim, 13 provided their written consent to participate in the study.

### Participant Characteristics

Thirteen participants who had been critically ill with COVID-19 during the first surge of the pandemic and had been admitted to a COVID-19-specific ICU, participated in the study. They were 32 to 78 years old (*M *= 60.15, *SD *= 12.24), five were women, and eight were men. Nine worked full-time, three were full-time pensioners and one was part-time retired. They had received respiratory treatment due to COVID-19 for 9 to 56 days (*Mdn *= 20) and either been intubated or received tracheostomy. During their hospitalization, they had also been transferred from other hospitals in the region. On average, the distance between the COVID-19-specific ICU and their homes was 167 km (range: 55–389 km).

### Ethical Considerations

All prospective participants received written and oral information about the study from the head of the COVID-19 ICU from which they were recruited. They were also informed that their participation was voluntary and that they could withdraw from the study at any time for any or no reason. All willing participants provided their written consent to participate in the study and were guaranteed confidentiality and anonymity in the presentation of the findings. The study was approved by the Swedish Ethical Review Authority (DNR: 2020-02805).

### Data Collection

Data were collected via individual interviews following a narrative approach, which in accordance with Mishler (1986) can create a condition of understanding the meanings that emerge during the interview. The interviews began with an open question “Can you please talk about your experiences of recovering from COVID-19,” in which the participants were given the possibility to freely narrate their experiences. Probing questions such as “Can you give an example?” and “How did you feel then?” were asked to encourage participants to elaborate on their answers. Asking such questions is important for understanding the meaning of a phenomenon, including recovery, from the perspective of the people experiencing it and for researchers to not take for granted what they mean. In accordance with the participants’ wishes, 10 interviews were conducted over the phone, whereas three were conducted face-to-face. The interviews lasted 35 to 80 min (*Mdn *= 55) and were audio-recorded and transcribed verbatim. One author (ÅE) conducted all interviews from July 2020 to March 2021, about 3 to 6 months after the participants had been discharged from the hospital.

### Data Analysis

As our study's aim was to elucidate meanings of recovery for people once critically ill with COVID-19, we analyzed the interview transcripts according to phenomenological hermeneutic interpretation, a method inspired by Paul Ricœur (1976) and further developed by Lindseth and Norberg (2004). Following that method, we sought a deeper understanding of the phenomenon under study through interpretation, which, according to Ricœur, lays bare the private meanings that are otherwise hidden in a text and makes them public.

Phenomenological hermeneutic interpretation consists of three phases: developing a naïve understanding, performing structural analysis, and developing a comprehensive understanding. The approach is not linear but involves moving between the whole text and its parts, from understanding to explanation and, in turn, from explanation to comprehension.

To develop a naïve understanding, all authors read the text several times to grasp its “meaning as a whole.” First author wrote the naïve understanding, in dialogue with the last author. This phase guided the structural analysis, in which the authors (first and last) divided the text into meaning units identified in light of our study's aim. The meaning units were subsequently condensed and, according to their similarities and differences, sorted into subthemes and, in turn, themes. To validate the identified subthemes and themes, we assessed them in light of our naïve understanding. All authors read and gave their input in this phase of the analysis process, in which we restrained our preunderstanding as specialist nurses and nursing researchers, to not make definite what is indefinite (Dahlberg et al., 2008). Last, we developed a comprehensive understanding from, and with the support of, our naïve understanding, structural analysis, and preunderstanding, as well as the literature. In that phase, the aim is to recontextualize the text and focus more on the future than the past, which, per Ricœur (1976) and Lindseth and Norberg (2004), means focusing on what the text talks about more than what it says. In that way, the comprehensive understanding can be revised, widened, and deepened. In that process, critical reflections among all authors were important for self-awareness about our preunderstanding, in order to keep us openminded while deepening it to support the most probable interpretation of the findings (Lindseth & Norberg, 2021).

## Findings

### Naïve Understanding

A strong willpower to recover from COVID-19 was salient among the participants. They struggled to get back on track by finding balance in their everyday lives. Although they expressed gratitude for having survived, contradictory feelings such as hopelessness and uncertainty continued to affect their sense of vitality. Bodily limitations forced them to gradually resume physical activities, creating feelings of frustration. Even so, their mental endurance had been strengthened by spending time in nature. They viewed returning to work as a way to normalize their lives and had longed for their colleagues. At the same time, they realized that not everyone understood how critically ill they had been. They reported that healthcare professionals had not asked them about how they felt upon returning home, which had led to feelings of abandonment and a perceived lack of support. To enable their recovery, they had wanted someone to talk to about their experiences and how they might deal with their feelings and lack of strength.

### Structural Analysis

The structural analysis resulted in one overarching theme—striving for balance in everyday life consisting of four subthemes: feeling willpower and gratitude; striving to regain strength; struggling for normalcy, and longing for support. The following subsections present the results of our structural analysis, accompanied with quotations from the interviews that we attribute to participants using pseudonyms.

### Striving for Balance in Everyday Life

#### Feeling Willpower and Gratitude

The participants described feeling a strong sense of willpower to recover from COVID-19. They had struggled to return home and looked forward to resuming their ordinary lives. Even though their lack of strength had led to feelings of insecurity, they described that engaging in the tasks of daily life had been a way toward recovery. Another way was to be in nature which was described as increasing their willpower to continue the struggle to recover and to gain relief from stressful thoughts. Their values, their preferences in life, and the meaning of life had changed after their illness, and they described being humbler in the face of what life had in store for them. They reported not putting forth as much effort toward trivialities as they had, before becoming critically ill:

Being out in nature purifies the soul—being in the woods and picking mushrooms. I also think that it dispels my thoughts. (Beatrice)

It's as if my whole mindset has changed. I more often think that everything will be OK; if I don’t find time to do things today, then I can do them tomorrow. This whole life carousel, to keep pace with it—it's like, after the illness, I see no need to rush. It's calmer now. (Oliver)

The participants additionally described wanting to understand the illness and why they had been stricken. They also described having become more sensitive since the illness. Ones who had read their ICU diaries appreciated the stories because it made their experiences with illness real instead of perpetuating the feeling that they had been fantasies. They described feeling grateful for having survived but became sad at times, and those mixed feelings were described as difficult to understand and too embarrassing to reveal to others. Thus, their hope for recovery had been interspersed with feelings of hopelessness:

I tell everyone that we need to be grateful for life, and that's why I feel so ashamed when I get sad, because I should be glad. It's hard to describe why I’m sad. I can’t tell my family about those feelings; I keep them to myself. (Charlotte)

#### Striving to Regain Strength

Participants described feeling eager and motivated to get back on track, both physically and mentally, after their illness. For some, physical exercise had led to exhaustion as they strove to engage in the same kind of activities as when they were healthy. Eventually, they had all learned their bodies’ limitations and described breathing to be the greatest challenge even when exercise also caused their bodies to ache. Those bodily limitations were described as frustrating, especially because the participants had the will to regain their strength at a faster pace:

I get frustrated when I’m panting while getting out of the boat or something like that. It's useless to be so skinny, but I still am. My diaphragm is half-paralyzed; I am in such bad shape. It's gone, so it must be rebuilt. (Christian)

The participants described daily activities as initially being challenging enough to allow them to regain their strength. They reported having lost an extreme amount of weight during their illness and that their muscle strength was limited; for that reason, simple physical exercise on the balcony, taking short walks near their homes, going fishing, and even picking berries had been sufficient activities for rebuilding muscle strength. The participants also reported setting goals to increase their strength. They enjoyed surviving and had learned that the recovery process would take longer time than they had initially expected. In that process, they had felt joy when their bodies responded to their efforts:

At the end of autumn, I’ll be hiking up in the mountains. If I can walk 800 meters, I’ll be happy. I can go up the path a bit behind so that civilization disappears, and I’ll see the mountains and the water where it gushes. It's so incredibly beautiful. (Robert)

#### Struggling for Normalcy

Participants described having been motivated and eager to resume their daily activities and work lives as soon as possible after returning home. Despite fatigue and a lack of strength, they had struggled for normalcy because they did not want to be a burden to others, but they had also longed for their colleagues. They still nurtured hope of becoming stronger in due time:

I thought that I should train and that I wouldn’t give up. I had no idea that it wouldn’t be better than this by now. I mean, I’ve never trained, but I decided that I should. (Oliver)

Upon first returning to work, the participants had experienced a low level of endurance, but their strength had progressively improved such that they could return to their full-time jobs and previous levels of activity. They reported getting stronger very quickly, and ones who had been prescribed aids such as walkers could eventually manage without them. Although they lacked full capacity, they had observed clear improvements in their strength. They had needed to adapt their daily lives to accommodate training as well as work and had scheduled training that they understood as being the most important. For some, it was a visit to a physiotherapist; for others, it was daily walks—for example, to and from work. Once back at home from work, all participants described needing to rest:

I haven’t entirely changed my lifestyle to be able to get back [to work], but I’ve tried. I’ve changed what needed to be changed, and I do the best that I can but not in any extreme way. (John)

Participants who worked described having received support from their employers—for example, extra leave to recover or support with contacting a doctor. Ones who worked in service-oriented professions and had become ill at work had reconsidered their situations and realized that they lacked support systems such as insurance when they, as employees, became ill at work. Participants also described having concerns about colleagues who did not understand that they had been critically ill and were still suffering from the effects. They also feared being reassigned due to their absence from work or that their own companies would collapse due to the COVID-19 pandemic.

#### Longing for Support

The desire to discuss the effects of COVID-19 with healthcare professionals was salient among the participants, who had various questions regarding memory loss, fatigue, and problems with concentration. They sought feedback from their X-rays due to indications that their lungs had been affected but in ways that they did not understand. Given how sick they had been, the participants indicated that their current physical and psychological disabilities were mild but nevertheless caused them to worry about whether they would be permanent. They thought that their recovery would have gone faster had they had the opportunity to talk to someone:

[I want] To have the chance to talk with someone, because I guess, or I feel, that my brain has been damaged. Especially in the beginning, I had problems with short-term memory, with reading, and with concentrating. I got so tired, but it slowly got better, and now I train my memory by reading, memorizing, and thereafter recalling. (Mattias)

Participants reported not being offered aftercare, which had made them feel abandoned. Despite being grateful for the care that they had received while critically ill, they felt that they should have at least been offered support upon returning home. They thus described how after care was underdeveloped and needed to be improved. Some participants were not contacted by healthcare professionals until up to 2 months after discharge. Such a long period without support had forced them to develop their own strategies to manage their daily lives:

There was no aftercare. Instead, it was like, “You’ve been really sick, we saved your life—which I am grateful for—but goodbye.” Then you’re outside the system, all alone. (John)

Participants who had taken the initiative to contact the healthcare center after returning home had felt accused of falling ill in COVID-19. They described feeling disappointed when no one asked them about how they felt, talked about their illness, or even read their medical records. They indicated that the healthcare professionals had insufficient knowledge about how to treat patients who have been critically ill with COVID-19 and the psychological suffering that it leads to. Some had met with a nurse or doctor whom they trusted, whereas others had received inappropriate comments from healthcare professionals:

One should be able to expect that they [doctors] have read your records, know what you’ve gone through, will ask how you’re feeling, and all that … but there was no questions or interest to listen to me … (Linda)

The participants described their relatives as a good source of support when healthcare had failed to offer aftercare. One participant described having a stubborn partner who had demanded support from healthcare when he became depressed. The participants reported needing to talk with someone other than their family members about their feelings, because doing so with family members would have been a source of strain. However, because healthcare professionals did not fill that role, the participants had felt that their questions, memories, and feelings were being ignored. They hoped that, in the future, healthcare professionals would have more knowledge about aftercare and support for individuals who were once critically ill with COVID-19.

### Comprehensive Understanding

We found that individuals who had been critically ill with COVID-19 strove for balance in their everyday lives and had the willpower to get back on track. In related research, Alexandersen et al. (2021) found that, for once critically ill patients, the motivation to return to ordinary life was health-promoting. Having been in a life-threatening situation increased their willpower, which, along with their inner strength, helped them despite limitations. Along those lines, Lundman et al. (2010) have defined *inner strength* as having the courage to continue striving to make life meaningful, endure, and deal with difficulties. Schaap et al. (2022) additionally found that an optimistic view on recovery was essential for individuals suffering from the post-illness effects of COVID-19. For those individuals, reassessing the importance of their own health and balance in life was one strategy used during the recovery process. In our study, participants described how they had reevaluated their outlook on life and had stop putting forth as much effort on keeping pace with their lives as before. They also expressed gratitude for having survived and being at peace with what had happened to them as a result of contracting COVID-19. This can be understood from Todres and Galvin (2010) writings, with reference to Martin Heidegger's philosophy, of dwelling-mobility, in which dwelling is a way of settling into the present and accepting things as they are, while mobility emphasizes the call of the future and a feeling of possibility. Here, the illness, that is, COVID-19 made the participants vulnerable as it caused an interruption in their everyday familiar life, which in accordance with Todres and Galvin can be seen as existential homelessness. By “letting-be” what has been, united with the energized flow of movement forward on future, offers a possibility for existential homecoming and further a feeling of wellbeing. Recovery is however a complex process, in which the concept itself according to Friedman (2021) can be misleading, as there is a distinguish between the bio-medical, first-person, and social perspectives. By that, the person can have recovered from the disease, as in this study, COVID-19 from a bio-medical perspective but experience illness, vulnerability, and suffering from the first-person perspective. Our interpretation is that participants on this study, despite of being discharged from hospital and considered as healthy, still suffered from unpredictability regarding their strength and understanding of what had happened when they became so ill. Navigating changes in one's life, requires understanding the situation and why one was stricken with adversity such as illness. Frank (2004) states that the patient should not be excluded from being a part of their treatment and healing. Instead, they should in accordance with Gadamer (2004) writings have the possibility to be offered a dialogue, in which they together with the healthcare could find a way to homecoming into their lifeworld. Todres et al. (2014) highlights the importance of caring for the patients “insiderness.” Healthcare professionals can be the one the patient can trust, the one who shows recognition to the patients’ needs and by that take the patient's vulnerability seriously. Such a lifeworld-led approach focuses on what the person can or can’t do in their everyday lives, aiming to help the person to do something that foresee their needs. In that light, even if patients have the willpower to recover, support from healthcare is important to promoting feelings of gratitude, wellbeing, and, in turn, the manageability of everyday life (Büssing et al., 2021). Following hospitalization, primary care is generally responsible for the aftercare of patients. However, as Schmidt et al. (2021) have shown in their narrative review, healthcare providers in primary care might need training in managing ICU survivors, especially survivors of COVID-19, in ways that afford them relevant support based on their needs. This is further stressed by Guo et al. (2022) where survivors of COVID-19 experienced great concerns about their prognosis and the exaggerated information from social media due to COVID-19 being novel and clear guidelines were lacking for treatment and care.

Our findings show that participants who had been critically ill with COVID-19 were eager to get back on track, mentally as well as physically, and tried to be as active as before illness. They felt frustrated by their lack of strength, which related to sometimes extreme weight loss and challenges with breathing. The attention that the body demands during illness can be understood in the light of Toombs’s (1993) ideas that such a body stands in opposition to the self and that a malfunctioning body needs to be considered in all aspects of life. As embodied subjects, we access the world by means of our body, meaning that we do not only have a body, but are our bodies. When healthy, daily activities are done without reflection regarding our bodily functions, meaning our embodied consciousness, is an action for what we can do. According to Merleau-Ponty (1996), all changes that occur in the body imply changes in the experience of being in the world. Illness represents a disability to be engaged in the world in a habitual way. As we are our bodies, illness does not only lead to a disintegration of our body, but also our self and the world. For recovery or healing as Friedman (2021) choose to call recovery from the first-person perspective, healthcare should not solely pay attention on the dysfunction of the biological body, but also take into consideration the disruption of the patients lived body (Toombs, 1993).

Participants in our study found that being in nature increased their willpower as well as relieved stressful thoughts. We refer this to the attention restoration theory in which Kaplan and Kaplan state that nature facilitates the persons’ spontaneous attention, meaning that one does not need to be concentrated on tasks or obligations from surrounding world. Instead, the person can when in spontaneous attention distance themselves from stressful thoughts, while providing a possibility for reflection about one's life (Kaplan, 1995). Nature can be a calming refuge (Johansson et al., 2023) and from Dahlberg (2007) writings we understand that one can feel companionship with nature, meaning that one can feel rest and stillness as well as joy and creativity despite of being lonely. In such loneliness which Dahlberg call a “good” loneliness, by us interpreted as solitude, one can feel at home. Engström and Alerby (2021) have shown that contact with nature, even when hospitalized, can heighten the sense of one's connectedness with nature, which can inform strategies for self-care upon being discharged from the hospital. Such self-care can be encouraged by nurses and other health care professionals for patients during their recovery processes.

In their struggle for normalcy, participants in our study were eager to resume their daily activities and return to work. This can be understood from Brough (2001) referring to Husserl's writing about temporality as our intentional awareness of time. Here, not by means of clock time, rather temporal modes of appearance as now, past and future. We live in our “now” from which we direct our attention to past and future. In illness, this can be distorted, and one can be stranded in the present, while the past is seen in a new sense, and the future which one has longed and planned for can be suspended. In accordance with Toombs (1993) such change in temporal significance can be experienced as a chaotic disturbance. One's sense of self is altered, but the self has a continuity even if the time is temporal, meaning that the continuous self can serve as a ground to restore one's changed life. From Brough (2001) writings it can be understood that the temporal horizon of future also can relieve hope as for example related to the participants in our study, who hoped for recovery in due time, meaning that their bodily temporality as weak and fatigued, will not go on endlessly. They further, longed for their colleagues, which can be related to Dahlberg (2007) who show the value of feeling belonging, to have a context to be a part of, which is possible in companionship with others for example at work, that is, colleagues who are available for companionship. All humans need to be connected, confirmed, and valued to value themselves. Such a context can contribute to recovery, health, and a feeling of wellbeing.

Our findings show that the lack of aftercare led to feelings of abandonment. Participants recovering from COVID-19 felt that health care professionals were not interested in them and lacked knowledge about the consequences of their illness. To achieve a balance between the effects of the illness and feeling well, individuals experiencing illness, or its effects need to be met with compassion and genuine interest (Thorne et al., 2004). To alleviate the patients suffering, Toombs (1993) state that attention should be paid not only to their sensory experience but also to their apprehension of the illness. Suffering increases with sole focus on physical anomalies. Attention should instead be on the patient's meaning of the illness. Healthcare professionals can never fully understand the meaning of the suffered illness of the patient, but need to seek to understand how the patient understands his/her illness, while also communicate their explanation model for the patient. By that, a shared understanding can be achieved (Toombs, 1993) and the patient can feel comfort (Thorne et al., 2004). Related to Buber (1997) writings the patient can also feel validated, which can facilitate internal growth that consequently supports the recovery process. In our study, participants developed their own strategies to manage their daily lives but feared falling ill again and had a wish to talk to someone about their experiences. To ignore patients’ needs is demoralizing (Frank, 2004) and leaves patients with a sense of being alone. In accordance with the tenets of person-centered care, patients should have the right to tell their stories and be supported (Ekman et al., 2011).

### Strengths and Limitations

To reveal possible meanings of recovery from critical illness with COVID-19, we chose to interpret the participants’ narrations by using phenomenological hermeneutic interpretation. The participants were willing to freely narrate their experiences during interviews, and probing questions were asked to minimize the risk of misunderstanding based on the interviewer's preunderstanding. Ten of the interviews were conducted by telephone. Even if face-to-face interviews should be the gold standard for data collection in qualitative research, Novick (2008) states that telephone interviews can allow participants to relax and more easily disclose sensitive information, which was the case on our study. Throughout the process of, we remained aware of our preunderstanding by engaging in critical peer discussions about how we understood the phenomenon under study (Ricœur, 1976). The interpretation presented herein is the one that we found to be most probable after considering and comparing other possible interpretations (Lindseth & Norberg, 2004). The narratives were rich and had depth in the variations of participants’ experiences, which is why we argue that they are the most significant experiences of recovery. The findings from this study cannot be generalized but can be transferred to other, similar situations, participants, and contexts (Lindseth & Norberg, 2004).

## Implications for Practice

Recovering from a critical, life-threatening illness can be overwhelming, especially if it is a lonely process, as it was for the participants in our study. There is a need for a structured plan for aftercare or at least follow-up calls for people who have suffered from critical illness due to COVID-19. To ensure a positive recovery process, we suggest, that people once critically ill with COVID-19 should have opportunities to share their stories about having the illness. In so doing, they gain the opportunity to understand the trajectory of their illness and their recovery process.

## Conclusions

In conclusion, meanings of recovery for people once critically ill with COVID-19, include a strive for balance in everyday life. For them, recovery seemed to be a complex process and despite having the willpower to return to their normal daily lives, they have to struggle to keep pace with others’ lives. Bodily limitations disabled them to be active as before, why they needed to seek for alternative ways of being in the world and participate in life. To understand what has happened and having the opportunity to reflect on personal fears and goals bare meaning for their recovery. As a part of the ill persons’ recovery process, healthcare professionals should seek to understand what the illness means for them, and also how they understand their illness. By that, they can find a mutual understanding with the person, and support them based on their needs.
